# Socioeconomic gradients in 24-hour movement patterns across weekends and weekdays in a working-age sample: evidence from the 1970 British Cohort Study

**DOI:** 10.1136/jech-2023-221726

**Published:** 2024-05-13

**Authors:** Joanna M Blodgett, David Bann, Sebastien F M Chastin, Matthew Ahmadi, Emmanuel Stamatakis, Rachel Cooper, Mark Hamer

**Affiliations:** 1 Institute of Sport, Exercise and Health, UCL, London, UK; 2 NIHR University College London Hospitals Biomedical Research Centre, London, England, UK; 3 Centre for Longitudinal Studies, Social Research Institute, UCL, London, UK; 4 School of Health and Life Sciences, Glasgow Caledonian University, Glasgow, UK; 5 Mackenzie Wearables Research Hub, Charles Perkins Centre, Faculty of Medicine and Health, The University of Sydney, Sydney, New South Wales, Australia; 6 School of Health Sciences, Faculty of Medicine and Health, The University of Sydney, Sydney, New South Wales, Australia; 7 AGE Research Group, Newcastle University, Newcastle upon Tyne, UK; 8 NIHR Newcastle Biomedical Research Centre, Newcastle upon Tyne, Newcastle upon Tyne, UK

**Keywords:** EDUCATION, SOCIAL CLASS, COHORT STUDIES, EPIDEMIOLOGY, EXERCISE

## Abstract

**Background:**

Socioeconomic differences in movement behaviours may contribute to health inequalities. The aim of this descriptive study was to investigate socioeconomic patterns in device-measured 24-hour movement and assess whether patterns differ between weekdays and weekends.

**Methods:**

4894 individuals aged 46 years from the 1970 British Cohort Study were included. Participants wore thigh-worn accelerometers for 7 days. Movement behaviours were classified in two 24-hour compositions based on intensity and posture, respectively: (1) sleep, sedentary behaviour, light-intensity activity and moderate-vigorous activity; and (2) sleep, lying, sitting, standing, light movement, walking and combined exercise-like activity. Four socioeconomic measures were explored: education, occupation, income and deprivation index. Movement behaviours were considered compositional means on a 24-hour scale; isometric log ratios expressed per cent differences in daily time in each activity compared with the sample mean.

**Results:**

Associations were consistent across all socioeconomic measures. For example, those with a degree spent more time in exercise-like activities across weekdays (10.8%, 95% CI 7.3 to 14.7; ref: sample mean) and weekends (21.9%, 95% CI 17.2 to 26.9). Other patterns differed markedly by the day of the week. Those with no formal qualifications spent more time standing (5.1%, 95% CI 2.3 to 7.1), moving (10.8%, 95% CI 8.6 to 13.1) and walking(4.0%, 95% CI 2.2 to 6.1) during weekdays, with no differences on weekends. Conversely, those with no formal qualifications spent less time sitting during weekdays (−6.6%, 95% CI −7.8 to –4.8), yet more time lying on both weekends (8.8%, 95% CI 4.9 to 12.2) and weekdays (7.5%, 95% CI 4.0 to 11.5).

**Conclusions:**

There were strong socioeconomic gradients in 24-hour movement behaviours, with notable differences between weekdays/weekends and behaviour type/posture. These findings emphasise the need to consider socioeconomic position, behaviour type/posture and the day of the week when researching or designing interventions targeting working-age adults.

**WHAT IS ALREADY KNOWN ON THIS TOPIC**

- There is conflicting evidence of the direction of association between socioeconomic position (SEP) and sedentary behaviour (SB) and physical activity (PA).
- Previous research has usually examined movement behaviours in isolation, often as total PA or SB time, without considering the day of the week or movement type.

**WHAT THIS STUDY ADDS**

- Results indicate clear socioeconomic gradients in 24-hour movement patterns, with marked differences between weekdays and weekends and movement behaviour type.
- Those with lower SEP spent more time standing, moving and walking and sat less during weekdays; conversely, these SEP differences were reversed on weekends.
- Those with higher SEP spent more time in combined exercise-like activities and less time lying down regardless of the day of the week.

**HOW THIS STUDY MIGHT AFFECT RESEARCH, PRACTICE OR POLICY**

- These findings provide novel insights into socioeconomic inequalities in movement behaviours and underscore the importance of considering both the day of the week and SEP when researching or designing interventions related to movement behaviours.

## Introduction

Socioeconomic gradients in health behaviours, such as physical activity (PA) and sedentary behaviour (SB), may contribute to the higher morbidity and mortality rates observed in socioeconomically disadvantaged groups.^1^ Consequently, it is important to understand how PA and SB patterns differ by socioeconomic position (SEP)^2^ in order to develop effective policies and interventions. SEP disparities are typically associated with lower PA levels^3 4^; both self-reported and device-measured data have repeatedly suggested that individuals with higher SEP engage in higher levels of leisure time PA.^5–8^ However, there is varied evidence as others report null findings^9–11^ or even inverse associations.^12 13^ Conflicting evidence also exists regarding SB, with higher levels of TV watching in those with lower SEP,^14 15^ while desk-based jobs are linked to higher SB in those from higher SEP.^16 17^ Alongside sex differences in occupation, leisure and household-based PA, weekly timing of movement behaviours is likely to play a role in these discrepancies. Activity patterns in individuals of working age are naturally constrained by working schedules (eg, traditional office hours and shift work),^18^ and therefore, socioeconomic patterning of SB and PA may differ by the day of the week.

Most research examining inequalities in PA has examined PA and SB behaviours in isolation or considered categorical phenotypes related to PA guidelines(eg, ‘active couch potato’ and ‘weekend warrior’^19 20^). This has provided an incomplete understanding of socioeconomic differences in movement behaviours, given the clear interdependence of behavioural and biological pathways underlying behaviours across the 24-hour day.^21^ Increasingly, PA epidemiologists have applied compositional approaches to investigate 24-hour movement behaviours, instead of isolated exploration of PA or SB.^21^ These approaches simultaneously consider the relative time spent in different movement types within a finite day,^21^ accounting for shared behavioural considerations and exploring potential implications of changes between pairs of behaviours (ie, replacing SB with varying activities).^22^

There is a need to better understand conflicting evidence in socioeconomic patterning of 24-hour movement behaviours to inform the development of new national and international guidelines using a 24-hour approach. Conflicting evidence could be due to differences in context (eg, occupation or leisure), movement or posture type (eg, sitting vs lying, walking vs running), or age (eg, children, working age and retired individuals). Thigh-worn accelerometers now provide improved postural identification, allowing greater sensitivity in detecting posture and activity types.^23^ Finally, the separation of weekend and weekday data enables an improved understanding of potential contextual factors and aids in improving implementation frameworks needed for targeted PA and SB interventions.

The aim of this descriptive study was to investigate socioeconomic patterning in 24-hour movement behaviours—including sleep, movement intensity and posture/activity type—and assess whether these patterns differ between weekdays and weekends. We used an age-homogenous sample of working-age individuals to eliminate differences in age-related patterning of habits and behaviours and explored any differences by sex.

## Methods

### Study sample

The 1970 British Cohort Study (BCS70) is an ongoing birth cohort comprising over 17 000 individuals born within a week of each other in April 1970 in England, Scotland and Wales.^24 25^ All births were eligible, including those in hospitals, homes and army facilities; the first questionnaire was completed by the midwife. At the most recent data collection wave at age 46 years, 8581 individuals participated; characteristics of those lost to follow-up have been described elsewhere.^24^ Individuals completed a computer-assisted questionnaire and interview biomedical tests and were asked to wear an accelerometer device for 7 days. Of 6562 individuals who consented to wear an accelerometer, 693 (10.6%) accelerometers were not initiated or not returned, data could not be downloaded for 858 (13.1%) and 117 (1.8%) had insufficient valid wear time (defined below), leaving data on 4894 for inclusion in analyses. Those who declined to wear accelerometers were more likely to be male, to be smokers, to report poorer health and to be obese.^26^

### Socioeconomic position

#### Education

At age 46 years, individuals reported any recent education or training they had completed since ages 38 and 42 years, which was compared with previous sweeps to determine participants’ highest academic qualification. Four categories were considered: none, up to General Certificate of Secondary Education (GCSE) level (typically age 16 years), up to A-levels or diploma (typically age 18 years) and degree or higher. Education is presented as the primary outcome throughout the paper as it is one of the more commonly used measures of SEP and is less susceptible to non-response bias and less likely to be affected by diseases that arise during adulthood.^2^ Ascertainment of secondary outcomes (occupational class, income and index of multiple deprivation (IMD)) is provided in online supplemental text 1.

10.1136/jech-2023-221726.supp1 Supplementary data

### 24-hour movement behaviours

Participants were invited to wear an activPAL3 microaccelerometer for 7 days, including during sleeping, bathing or swimming activities.^26^ Research nurses waterproofed and fitted the device on the midline anterior aspect of the upper thigh during the clinic visit. Data were processed using ActiPASS V.1.32, a validated software that can identify non-wear, sleep, and activity and posture types.^27 28^ The average time spent in movement behaviours was classified in two 24-hour compositions. Composition 1 captured a traditional 4-part day and consisted of sleep, SB (lying and sitting), light-intensity PA (LIPA) and moderate-vigorous PA (MVPA). Composition 2 comprised sleep, lying, sitting, standing, moving, walking and combined exercise-like activities.^27–29^ We combined running, cycling and inclined walking—previously categorised as high-intensity activities^22^—as a single category of exercise-like activities due to the small volumes of each component. Participants with 1+ weekday and 1+ weekend day (≥20 hours/day, ≥1 walking period and >0 min of sleep) were included in the analysis (n=4894). Although there is no established minimum wear time for 24-hour data, previous sensitivity analyses—including in this sample—suggest that results do not change when requirements were increased to 3+ days with 23+ hours/day of valid wear time.^22^

### Statistical analysis

Absolute time spent in each movement behaviour for weekdays and weekend days were described. We defined composition as the proportion of time spent in each behaviour (ie, 4-part or 7-part composition). For weekdays and weekend days, we calculated mean time (rescaled such that behaviours sum to 24 hours) for each educational group. Using sex-adjusted multivariate analysis of covariance (MANCOVA), we examined how movement behaviours differed across educational groups. Next, we described how time spent in each behaviour differs compared with the overall sample mean using isometric log ratio differences to express per cent differences; we derived bootstrap (n=1000) percentile confidence intervals for these differences.^30^ We repeated the above analyses for occupational class, income and IMD. Finally, we carried out additional analyses of sex-stratified models (decided a priori^10 13^) and investigated the characteristics of those who participated in the age of 46 years wave but had missing accelerometer data.

## Results

### Sample characteristics

A total of 4894 individuals were included in analyses, of which 25.7% of individuals had no formal qualifications and 28.4% had a university degree or higher (table 1). Nearly half of the sample (47%) reported having a lower managerial/intermediate occupation, with ~15% falling under each routine/semiroutine or small employer/lower supervisory. Median weekly income was £692 per week (Q1, £378, and Q3, £1036). Approximately a quarter of individuals were in the lowest four deciles of IMD (27.0%), 21% in deciles 5–6, 25% in deciles 7–8 and 26.9% in deciles 9–10. Men were more likely to have no qualifications than women (28.9% vs 22.3%) and be in a lower IMD decile (49.7% vs 46.6% deciles 1–6), yet they were more likely to have a professional/managerial role (22.5% vs 11.6%) and higher income (29.0% vs 24.6% in the highest quartile; online supplemental table 1).

**Table 1:** Characteristics of included sample (n=4894)

Socioeconomic position, n(%)		
Highest level of education attained (age 46 years)		
No formal qualifications	1242 (25.7%)	
Up to GCSE level	1499 (31.0%)	
Up to A-levels or diploma	720 (14.9%)	
Degree or higher	1373 (28.4%)	
Occupational class (age 46 years)		
Routine/semiroutine	704 (16.3%)	
Small employer/lower supervisory	768 (17.8%)	
Lower managerial/intermediate	2031 (47.0%)	
High professional/managerial	815 (18.9%)	
Income (age 46 years)		
Lowest quartile (<£378 /week)	996 (21.9%)	
Low-middle quartile (£378-£692 /week)	1161 (25.6%)	
Middle-high quartile (£693–1036/week)	1174 (25.9%)	
Highest quartile (>£1036 /week)	1211 (26.7%)	
Index of Multiple Deprivation (age 46 years)		
Deciles 1–4 (most deprived)	1318 (27.0%)	
Deciles 5–6	1026 (21.0%)	
Deciles 7–8	1228 (25.1%)	
Deciles 9–10 (least deprived)	1314 (26.9%)	
Movement behaviours*, mean±SD		
	**WEEKDAY**	**WEEKEND**
Composition 1—based on intensity:		
Sleep (hours/day)	7.0 (±1.2)	7.7 (±1.6)
Sedentary behaviour (hours/day)	10 (±2.2)	9.2 (±2.1)
Light activity (hours/day)	4.7 (±1.8)	4.7 (±1.7)
Moderate-vigorous physical activity (hours/day)	1.3 (±0.56)	1.3 (±0.64)
Composition 2—based on activity or posture type		
Sleep (hours/day)	7.0 (±1.2)	7.7 (±1.6)
Lying (hours/day)	1.6 (±1.2)	2.0 (±1.5)
Sitting (hours/day)	8.5 (±2.4)	7.2 (±2.2)
Standing (hours/day)	3.2 (±1.3)	3.2 (±1.3)
Moving (hours/day)	1.3 (±0.59)	1.3 (±0.54)
Walking (hours/day)	1.4 (±0.55)	1.3 (±0.59)
Combined exercise-like activity (hours/day)†	0.18 (±0.18)	0.20 (±0.30)

*Total time may not sum to 24 hours as mean wear time was 22.9±1.1 hours/day.

†Equivalent to 11±11 min and 12±18 min on weekdays and weekends, respectively.

GCSE, General Certificate of Secondary Education.

Average time spent in LIPA (4.7±1.8 vs 4.7±1.7 hours per day) or MVPA (1.3±0.56 vs 1.3±0.64 hours per day) was consistent across weekdays and weekend days; participants slept longer on weekends (7.7±1.6 vs 7.0±1.2 hours per day) and were more sedentary during weekdays (10.0±2.2 vs 9.2±2.1 hours per day; table 1, Composition 1). There were no differences in standing, moving, walking or combined high-intensity activity; however, individuals spent more time lying (2.0±1.5 vs 1.6±1.2 hours per day) on the weekend and more time sitting during the weekday (8.5±2.4 vs 7.2±2.2 hours per day; table 1, Composition 2).

### Composition 1: sleep, SB, LIPA, MVPA

**Figure 1:**
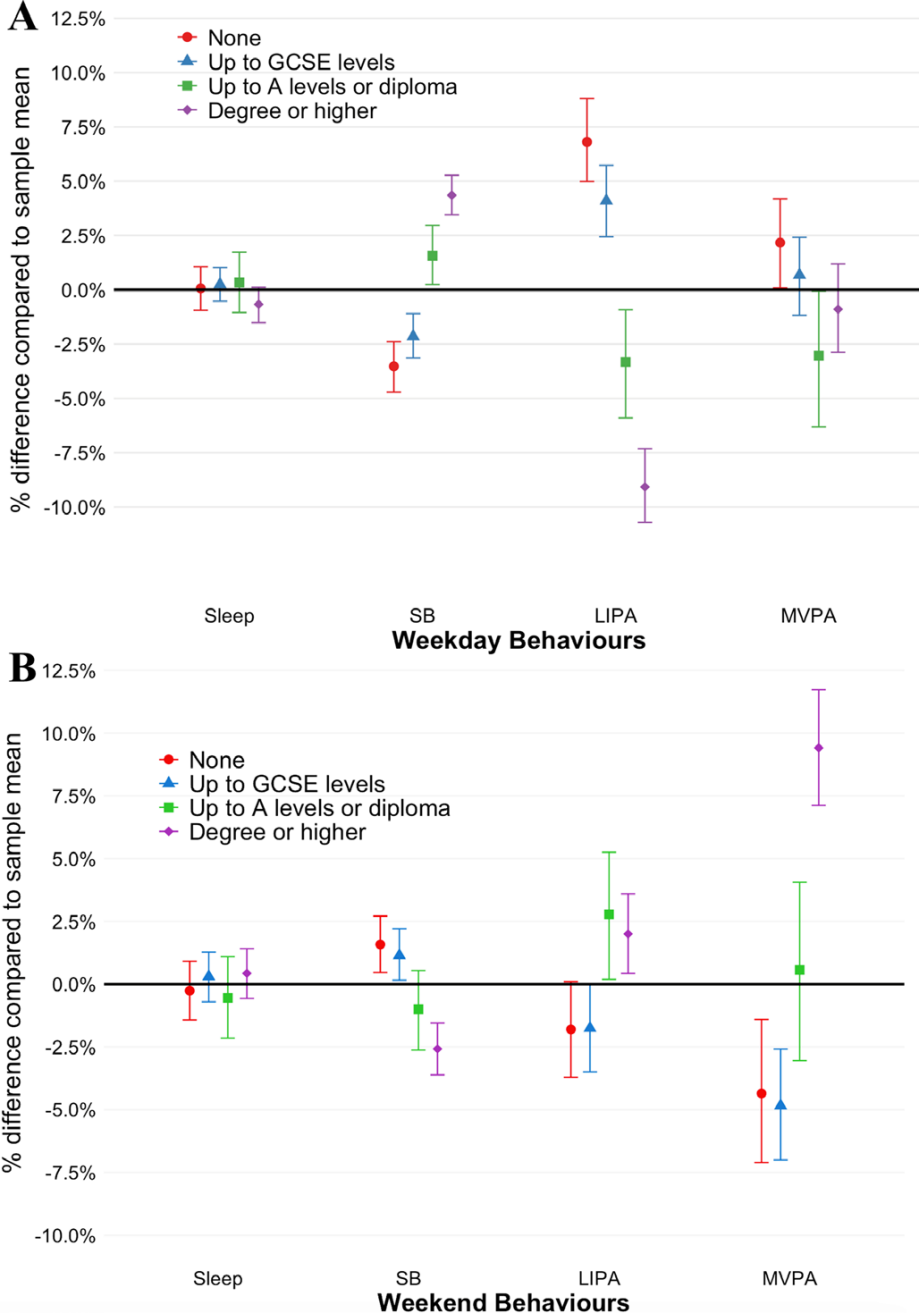
Per cent differences in daily movement between education groups and the sample mean on (A) weekdays and (B) weekends; derived with the log ratio of geometric mean values for sleep, sedentary behaviour (SB), light-intensity physical activity (LIPA) and moderate-vigorous intensity physical activity (MVPA), with bootstrap 95% CIs. GCSE: General Certificate of Secondary Education.

There was an overall association between each SEP indicator and Composition 1 for weekdays and weekend days (p<0.001). Figure 1 provides per cent differences in daily time spent in each behaviour for each education group compared with the overall sample mean. As patterns were similar across each outcome, education is primarily described in text; results are replicated in supplementary materials for occupational class, income and IMD (online supplemental figures 1–3). There was no difference in time spent sleeping by educational group for either day type. During weekdays, there was a graded association between education level and sedentary time; for example, compared with the overall sample, individuals with a degree or higher spent 4.3% (95% CI 3.4% to 5.4%) more time in SB, while those with no qualifications spent 3.6% (−4.7% to –2.6%) less time. During weekends, the opposite pattern was observed as individuals with higher education spent less time sedentary (degree:−2.7% (−3.6% to –1.5%)) and those with no qualifications spent more (1.5% (0.4% to 2.7%)).

Inequalities in activity patterns are also contrasted by the day of the week (figure 1). During weekdays, those with no formal qualifications and those with GCSE levels or below spent 6.8% (95% CI 5.1% to 8.8%) and 4.1% (2.5% to 5.8%) more time in LIPA, while those with A-levels/diploma and those with a degree spent 3.5% (−5.9% to –1.0%) and 9.2% (–10.8% to –7.4%) less time in LIPA (ref: overall sample). There was a similar, although weaker association for MVPA. Conversely, during the weekends, those with greater educational attainment spent more time in LIPA (none, −1.9% (95% CI –2.7% to 0.1%); GCSE, −1.7% (−3.4% to –0.0%); A-levels, 2.8% (0.2% to 5.2%); and degree or higher, 2.0% (0.4% to 3.6%)) and in MVPA (none, −4.3% (−7.1% to –1.5%); GCSE, −4.8% (−7.0% to –2.6%); A-levels: 0.5% (–3.0% to 4.0%); degree or higher, 9.3% (7.0% to 11.6%)).

Near identical associations were observed across other SEP indicators (online supplemental figures 2 and 3), although effect sizes were smaller for IMD and larger for occupation during weekdays. For example, there was little difference in SB time across IMD quartiles, despite clear trends for the other SEP indicators. Conversely, some per cent differences were twice the size for occupational class compared with education. For example, during weekdays, those in the lowest occupational classes engaged in ~20% more LIPA (vs ~7% in lowest educational classes), whereas the highest occupational class engaged in ~20% less (vs ~8% in highest educational class) (online supplemental figure 2A).

### Composition 2: sleep, lying, sitting, standing, moving, walking, combined exercise-like activity

There were also associations between each SEP indicator and the 7-part composition (p<0.001). Time spent in movement behaviours differed by educational attainment for all behaviours except sleep during the weekdays and for three behaviours during the weekends (lying, walk and combined exercise-like activity).

**Figure 2:**
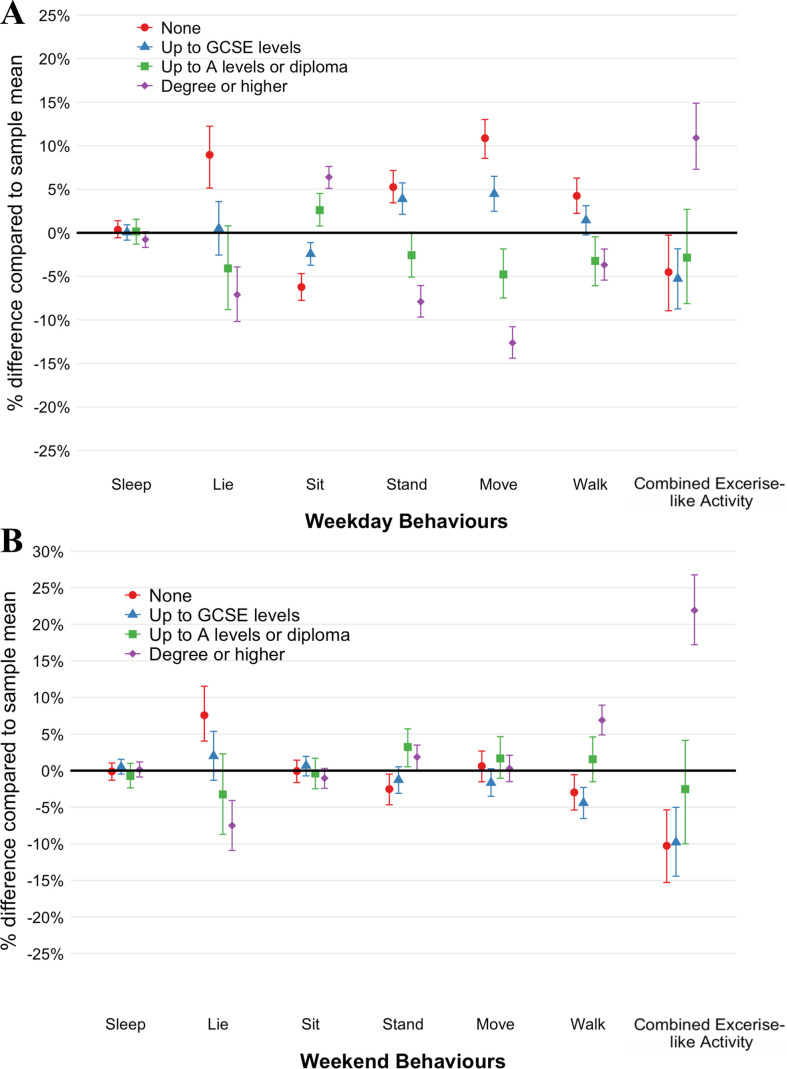
Per cent differences in daily movement between education groups and the sample mean on (A) weekdays and (B) weekends; derived with the log ratio of geometric mean values for sleep, lying, sitting, standing, moving, walking and combined exercise-like activity, with bootstrap 95% CIs. GCSE: General Certificate of Secondary Education.

During both weekdays and weekend days, those with lower education spent more time lying than those with higher educational attainment (figure 2). For example, compared with the overall sample, those with no qualifications spent 8.8% (95% CI 4.9% to 12.2%) and 7.5% (4.0% to 11.5%) more time lying on weekdays and weekend days, respectively. This dose-response association was observed in the opposite direction for sitting during the week, with increasing levels of sitting at higher education levels (none, −6.6%(95% CI −7.8% to –4.8%); GCSE levels, −2.6% (−3.8% to –1.2%); A-levels (2.6% (0.7% to 4.6%); or degree or higher (6.4% (5.0% to 7.5%)). There were no differences in sitting time during the weekends (figure 2B).

Standing, moving and walking exhibited similar patterns of association during the weekday; those with no formal qualifications spent more time (5.1% (95% CI 2.3% to 7.1%), 10.8% (8.6% to 13.1%) and 4.0% (2.2% to 6.1%), respectively), and those with a degree or higher spent less time(−8.2% (−9.9% to –6.3%), −13.0% (−14.7% to −11.0%) and −4.0% (−5.8% to –2.1%), respectively) in these activities. There were minimal differences between groups during the weekends, with evidence that those with more education spend more time walking (degree or higher: 6.9% (95% CI 4.9% to 9.0%)) than other groups. Finally, those with a degree or higher spent substantially more time in combined exercise-like activity than the rest of the sample across both weekdays (10.8% (95% CI 7.3% to 14.7%)) and weekends (21.9% (17.2% to 26.9%)).

As in Composition 1, similar patterns were observed for occupational class, income and IMD (online supplemental figures 4–6). However, there was little difference in sitting, standing or walking during the week by IMD quartile, with more sitting, less moving or walking in those living in more deprived areas during the weekends.

### Additional analyses

Associations were similar when stratified by sex (online supplemental figures 7 and 8), with some indication of slightly larger effect sizes in men. Those with missing accelerometer data were more likely to be male and have lower educational attainment (eg, 33.2% vs 25.7% no qualifications), lower occupational class, lower income and living in more deprived areas (online supplemental table 2).

## Discussion

We found clear socioeconomic gradients in 24-hour movement patterns in individuals aged 46 years, with marked differences between weekdays and weekends. Compared with higher SEP groups (ie, higher educational qualifications, occupational class, income or living in a lower deprivation area), those with lower SEP spent more time standing, moving and walking and sat less during weekdays; these SEP differences were reversed on weekends. Those with higher SEP spent more time in combined exercise-like activities and less time lying regardless of the day of the week. These findings provide novel insights into socioeconomic inequalities in movement behaviours and underscore the importance of considering SEP and weekdays when researching or designing interventions related to movement behaviours.

The novelty of this study is the consideration of the 24-hour composition and exploration of more nuanced posture (eg, sitting and lying) and activity (eg, standing, moving, walking and exercise) classifications. Findings address an important gap in the literature by reconciling conflicting evidence of associations between SEP and device-measured SB or PA.^4–7^ We observed more lying time in those with lower SEP, regardless of the day of the week, and higher sitting time in those with higher SEP during the week. This is likely due to occupational sitting patterns, with individuals from higher SEP more likely to have desk jobs.^17^ While context was not assessed in our study, we hypothesise that lying may be more consistent with television watching, video gaming or mobile phone use,^31^ which as self-reported evidence suggests is frequent among lower SEP groups.^14 15^ It may also reflect restricted opportunities to engage in leisure-time PA, due to economic or environmental factors.^32 33^

Different patterns emerged depending on the activity type and intensity and the day of the week. Individuals from lower SEP spent more time standing, moving and walking during weekdays, reflecting the increased likelihood of manual-based work in lower socioeconomic groups.^34^ Conversely, on weekends, differences in LIPA were negligible, with some evidence of less walking among those from lower socioeconomic groups. Lower walking time, combined with increased lying time on the weekends, might suggest that individuals in manual jobs prioritise rest instead of engaging in light leisure-time PA. Moreover, those from higher socioeconomic groups engaged in more high-intensity PA regardless of the day of the week. This may reflect greater participation in structured leisure-time PA and align with evidence suggesting that educational attainment is associated with greater engagement in health-promoting PA during leisure time.^6^ These differences contrast with the negligible differences observed in MVPA during weekdays, which are likely due to both occupational-based moderate-intensity activity such as fast walking and leisure-time high-intensity combined activities being captured.

Opposing patterns of weekday and weekend activity may reflect differences in occupational and leisure-time PA. This is supported by the stronger associations observed when examining occupational or income differences, which are more strongly correlated with occupational activity, compared with educational differences. This is also consistent with several reviews suggesting assumed positive associations between SEP and PA are mainly driven by leisure-time PA.^6 35 36^ We add to the evidence by suggesting an inverse association between occupational-based PA and SEP, although evidence combining self-reported data with objective assessments is needed.

### Implications

Our results have implications for national and international PA guidelines, as well as those designing interventions targeting SB or PA. Universal movement recommendations may be inadequate due to socioeconomic disparities in behaviours and financial, health and personal circumstances.^34^ Guidelines and interventions could instead be tailored to target samples and consider broader implications of how to effectively reach those in greatest need. There is a need to consider differences in SEP, occupation or leisure context, movement/posture (eg, lying vs sitting and exercise-like activities vs LIPA) and weekday. This may include interventions aimed to increase leisure-time PA on non-working days or standing-based interventions for those who spend most of their workday sitting.

The recommended 2.5 hours per week of MVPA may be challenging to achieve for working-age individuals with physically demanding jobs; occupational PA may not yield the same cardiovascular benefits as leisure-time PA.^37^ Personalised activity guidance could consider SEP and occupational demands and target communities, employers and local government to address wider socioeconomic disparities. The largest per cent differences were observed for high-intensity activities; however, the absolute time spent in MVPA (eg, ~1–5 hours per day) or specific exercise-like activities (~10–15 min per day) is a relatively small fraction compared with sedentary time (~9–10 hours per day). However, high-intensity activity demonstrates the strongest associations with health outcomes^21 22^ and therefore represents a feasible and efficient target for daily change. Evidence from an international consortium suggests that ~3–4 min per day of sedentary time replaced by MVPA could improve cardiometabolic health markers.^22^

The opposing socioeconomic gradients across weekdays and weekends suggest a need to consider the day of the week when analysing device-measured movement data. Although there are some recommendations when deriving summary variables (eg, 2+ weekends and 3+ weekdays or 1+ weekend day^38 39^), criteria can shift to 1+ or 4+ days due to competing desires to maximise sample size. Failure to consider the day may result in biased estimates of movement behaviour; this is applicable both to describing inequalities in these behaviours and to yielding accurate overall measures of behaviour, given established socioeconomic differences in wear time.^40^ Opposing associations between SEP and PA/SB by the day of the week highlight that it may not be appropriate to give equal weight to weekend days and weekdays to derive a single summary indicator of mean daily time. Future studies could report on the distribution of activity captured across weekdays and weekends and explore how associations with disease and mortality outcomes are sensitive to this distribution.

### Strengths and limitations

Key strengths include the large age-homogenous sample of working-age individuals, device-measured assessment of 24-hour behaviours and the ActiPASS algorithm to identify movement behaviours (ie, sitting, lying, standing, moving, walking, cycling and running). Limitations include the lack of movement behaviour context, potential underestimation of results due to missing accelerometer data in those from lower SEP, misclassification of some behaviours (eg, sleeping vs lying and LIPA vs MVPA) and activity types (eg, swimming and vigorous lifting). This study was descriptive in nature; future studies must extend findings to better investigate associations between SEP and movement behaviours. This includes further exploration of early life SEP, causal pathways between SEP, movement behaviours and health outcomes, and examining how SEP across life influences movement. Further research should also explore how these patterns may change across individuals with different working or personal circumstances (eg, shift work, retirement and childcare).

## Conclusions

Our study provides insight into the complex relationships between SEP and PA/SB patterns, revealing opposing patterns between weekdays and weekends. Those from lower SEP groups had higher movement during the weekday and lower movement during the weekend, although socioeconomic inequalities in exercise-specific activities were observed regardless of the day of the week. Findings highlight the importance of considering SEP, movement or posture type, and the day of the week when investigating or intervening in PA and SB. By recognising this, researchers and practitioners can tailor approaches to address the specific needs of diverse populations, ultimately promoting healthier lifestyles and narrowing socioeconomic health disparities.

## Data Availability

Data are available in a public, open access repository. The BCS70 datasets are publicly available in the UK Data Archive repository: BCS70 https://discover.ukdataservice.ac.uk/series/?sn=200001.
