# Supplementary material for: Socioeconomic gradients in 24-hour movement patterns across weekends and weekdays in a working-age sample: evidence from the 1970 British Cohort Study
Source: J Epidemiol Community Health. 2024 May 13;78(8):515–21. doi: 10.1136/jech-2023-221726 (PMC11287567; doi:10.1136/jech-2023-221726)
Supplement: Supplementary data [file jech-2023-221726supp001.pdf]

Supplementary Text 1. Ascertainment of secondary socioeconomic position outcomes (occupational class, income, index of multiple deprivation)

Occupational class**:** At age 46, individuals also reported their job title and provided a description of what they did in this role. Employment types were coded using the National Statistics Socioeconomic Classifications of Occupations (ONS, 2010) and considered in four categories: routine/semi-routine, small employer/lower supervisory, lower managerial/intermediate, high professional/managerial.

Income**:** At age 46, individuals were asked to report their take-home pay. Computer-assisted interviewing encouraged individuals to consult a most recent payslip, and specified that take-home pay was the amount paid after deductions for tax, national insurance, pension, union dues and other. Total weekly pay was derived for each participant and divided into four quartiles: <£378/week, £378-£692/week, £693-£1036/week or >£1038/week.

Index of Multiple Deprivation: The Index of Multiple Deprivation (IMD) is an official measure of relative deprivation of neighbourhoods in England, and is derived from seven domains: low income, unemployment, education, health, crime, barriers to housing and services and living environment (Noble et al., 2006). It is commonly presented as deciles – where 1 represents the most deprived neighbourhoods and 10 represents the least deprived across all of the UK. For consistency with the four-category structure of the other SEP indicators, four quartiles were considered: most deprived (deciles 1-4, 5-6, 7-8, 9-10).

References:

Office of National Statistics, 2010. The National Statistics Socio-economic classification (NS-SEC). Available at: https://www.ons.gov.uk/methodology/classificationsandstandards/otherclassifications/thenationalstatisticssocioeconomicclassificationnssecrebasedonsoc2010

Noble M, Wright G, Smith G, Dibben C. Measuring multiple deprivation at the small-area Level. Environment and Planning A. 2006;38:169–185.

Supplementary Table 1. Descriptive characteristics of analytical sample by sex

	Male (n=2268)	Female (n=2626)
**SOCIOECONOMIC POSITION, n(%)**		
**Highest level of education attained (Age 46)**		
None	656 (28.9 %)	586 (22.3 %)
Up to GCSE levels	669 (29.5 %)	830 (31.6 %)
Up to A levels or diploma	287 (12.7 %)	433 (16.5 %)
Degree or higher	619 (27.3 %)	754 (28.7 %)
**Occupational Class (Age 46)**		
Routine/Semi-routine	318 (14.0 %)	386 (14.7 %)
Small employer/lower supervisory	514 (22.7 %)	254 (9.7 %)
Lower managerial/intermediate	786 (34.7 %)	1245 (47.4 %)
High professional/managerial	510 (22.5 %)	305 (11.6 %)
**Income (Age 46)**		
Lowest quartile (<£378/week)	435 (20.4%)	561 (23.3%)
Low-middle quartile (£378-£692/week)	526 (24.7%)	635 (26.4%)
Middle-high quartile (£693-1036/week)	553 (25.9%)	621 (25.8%)
Highest quartile (>£1036/week)	618 (29.0%)	593 (24.6%)
**Index of Multiple Deprivation (Age 46)**		
Deciles 1-4 (most deprived)	643 (28.4%)	675 (25.8%)
Deciles 5-6	482 (21.3%)	544 (20.8%)
Deciles 7-8	546 (24.1%)	682 (26.0%)
Deciles 9-10 (Least deprived)	595 (26.3%)	719 (27.4%)
**MOVEMENT BEHAVIOURS (mean ±SD)**		
**Composition 1**		
**WEEKDAY**		
Sleep (hours/day)	6.7 (±1.2)	7.2 (±1.2)
Sedentary Behaviour (hours/day)	10 (±2.2)	9.8 (±2.1)
Light-Intensity PA (hours/day)	4.6 (±1.8)	4.8 (±1.7)
Moderate to Vigorous PA (hours/day)	1.3 (±0.58)	1.3 (±0.54)
**WEEKEND**		
Sleep (hours/day)	7.4 (±1.7)	8.0 (±1.5)
Sedentary Behaviour (hours/day)	9.5 (±2.2)	9.0 (±2.0)
Light-Intensity PA (hours/day)	4.6 (±1.8)	4.7 (±1.7)
Moderate to Vigorous PA (hours/day)	1.2 (±0.69)	1.3 (±0.60)
**Composition 2**		
**WEEKDAY**		
Lying (hours/day)	1.8 (±1.4)	1.5 (±1.1)
Sitting (hours/day)	8.7 (±2.5)	8.3 (±2.3)
Standing (hours/day)	3.1 (±1.3)	3.4 (±1.3)
Moving (hours/day)	1.3 (±0.64)	1.2 (±0.54)

Walking (hours/day)	1.3 (±0.58)	1.4 (±0.52)
Combined Exercise-like Activities (hours/day)	0.22 (±0.22)	0.17 (±0.16)
**WEEKEND**		
Lying (hours/day)	2.2 (±1.6)	1.9 (±1.3)
Sitting (hours/day)	7.3 (±2.3)	7.1 (±2.1)
Standing (hours/day)	3.0 (±1.3)	3.3 (±1.3)
Moving (hours/day)	1.3 (±0.57)	1.2 (±0.52)
Walking (hours/day)	1.3 (±0.62)	1.3 (±0.57)
Combined Exercise-like Activities (hours/day)	0.23 (±0.38)	0.17 (±0.23)

GCSE: General Certificate of Secondary Education

Supplementary Table 2. Descriptive characteristics of analytical sample by sex

	Included sample (n=4894)	Participated in age 46 wave, but no accelerometer data (up to n=3687)
**Sex**		
Male	2268 (46.3%)	1886 (51.2%)
Female	2626 (53.7%)	1801 (48.9%)
**SOCIOECONOMIC POSITION, n(%)**		
**Highest level of education attained (Age 46)**		
None	1242 (25.7%)	1197 (33.2%)
Up to GCSE levels	1499 (31.0%)	1161 (32.3%)
Up to A levels or diploma	720 (14.9%)	453 (12.6%)
Degree or higher	1373 (28.4%)	799 (22.1%)
**Occupational Class (Age 46)**		
Routine/Semi-routine	704 (16.3%)	640 (21.0%)
Small employer/lower supervisory	768 (17.8%)	589 (19.3%)
Lower managerial/intermediate	2031 (47.0%)	1264 (41.4%)
High professional/managerial	815 (18.9%)	562 (18.4%)
**Income (Age 46)**		
Lowest quartile (<£378/week)	435 (20.4%)	561 (23.3%)
Low-middle quartile (£378-£692/week)	526 (24.7%)	635 (26.4%)
Middle-high quartile (£693-1036/week)	553 (25.9%)	621 (25.8%)
Highest quartile (>£1036/week)	618 (29.0%)	593 (24.6%)
**Index of Multiple Deprivation (Age 46)**		
Deciles 1-4 (most deprived)	643 (28.4%)	675 (25.8%)
Deciles 5-6	482 (21.3%)	544 (20.8%)
Deciles 7-8	546 (24.1%)	682 (26.0%)
Deciles 9-10 (Least deprived)	595 (26.3%)	719 (27.4%)

GCSE: General Certificate of Secondary Education

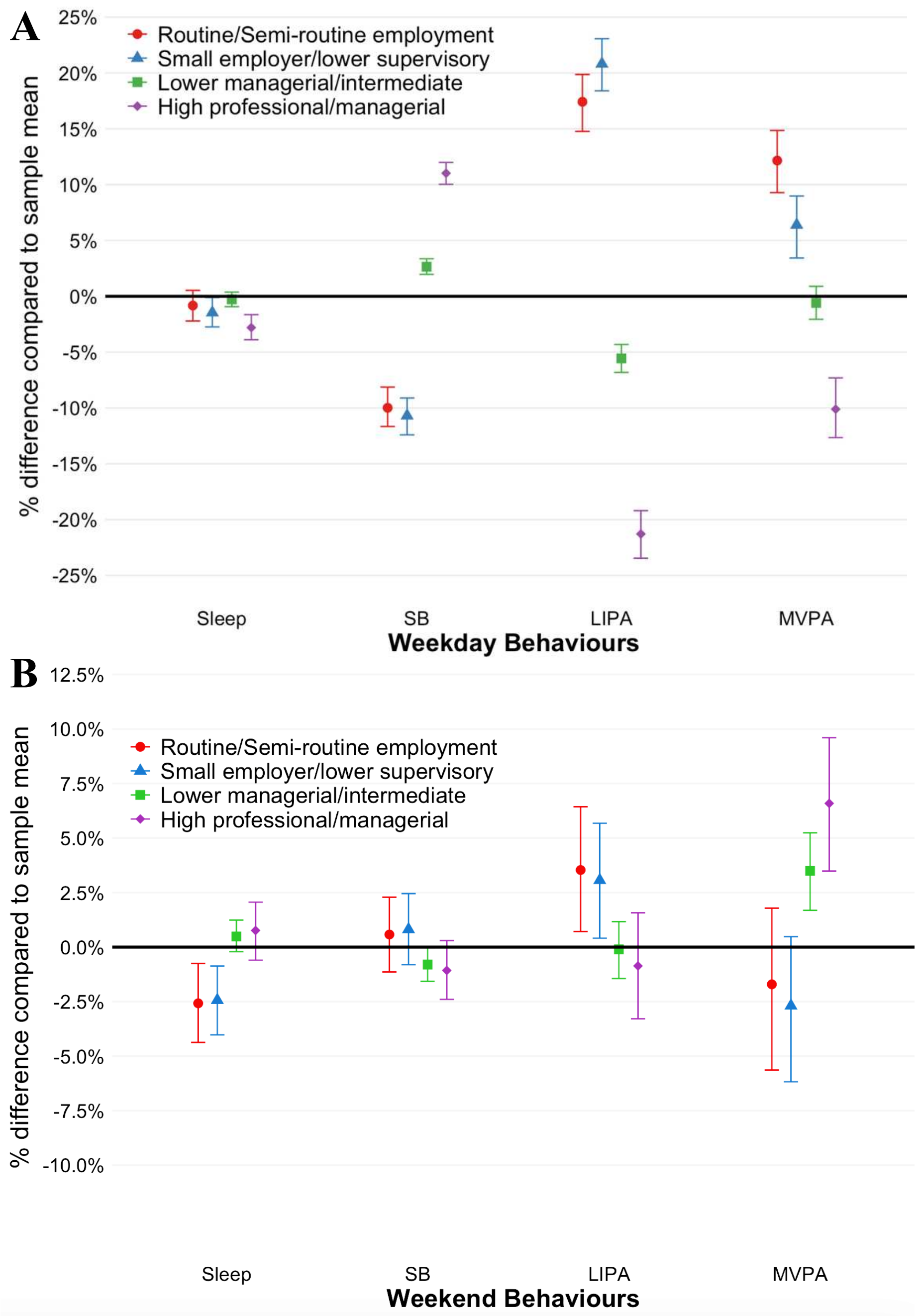

Supplementary Figure 1. Percent differences in daily movement between **occupational classes** compared to the sample mean on **A)** Weekdays and **B)** Weekends; derived with the log-ratio of geometric mean values for sleep, sedentary behaviour, light-intensity physical activity and moderate to vigorous intensity physical activity, with bootstrap 95% percentile confidence intervals.

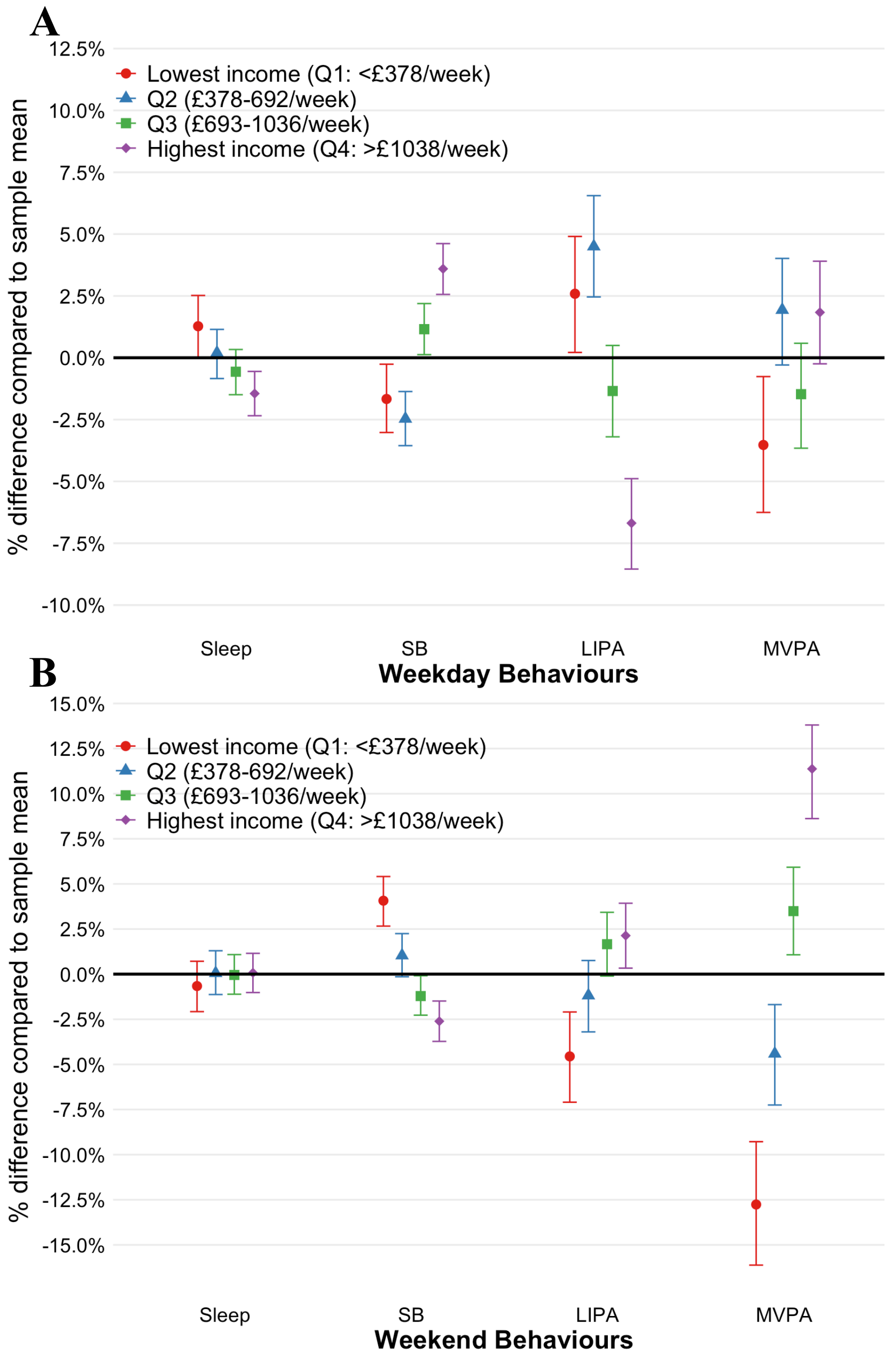

Supplementary Figure 2. Percent differences in daily movement between **income quartiles** compared to the sample mean on **A)** Weekdays and **B)** Weekends; derived with the log-ratio of geometric mean values for sleep, sedentary behaviour, light-intensity physical activity and moderate to vigorous intensity physical activity, with bootstrap 95% percentile confidence intervals.

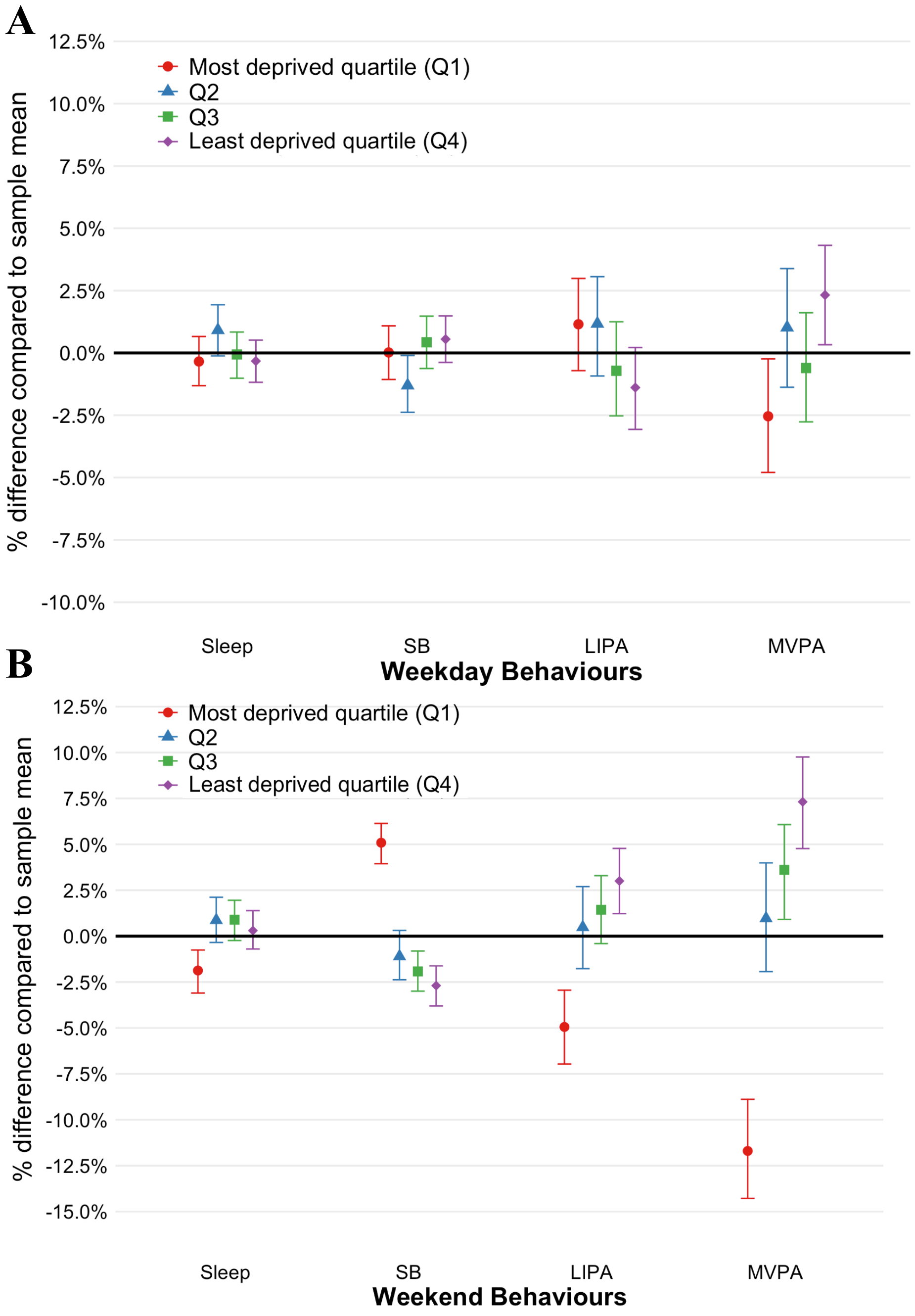

Supplementary Figure 3. Percent differences in daily movement between **IMD quartiles** compared to the sample mean on **A)** Weekdays and **B)** Weekends; derived with the log-ratio of geometric mean values for sleep, sedentary behaviour, light-intensity physical activity and moderate to vigorous intensity physical activity, with bootstrap 95% percentile confidence intervals.

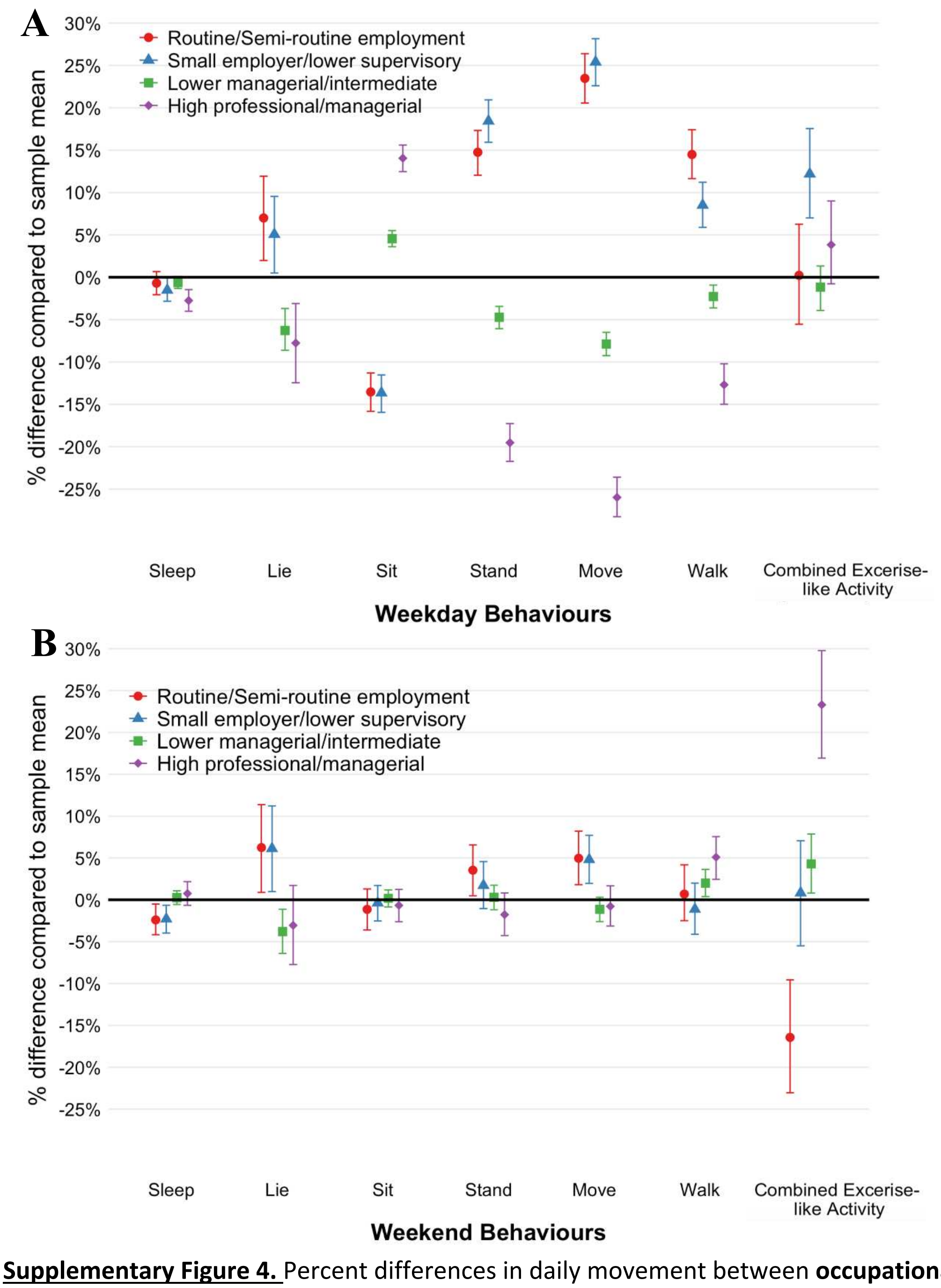

__Supplementary Figure 4.__ Percent differences in daily movement between **occupational classes** compared to the sample mean on **A)** Weekdays and **B)** Weekends; derived with the log-ratio of geometric mean values for sleep, lying, sitting, standing, moving, walking, combined-high intensity physical activity, with bootstrap 95% percentile confidence intervals.

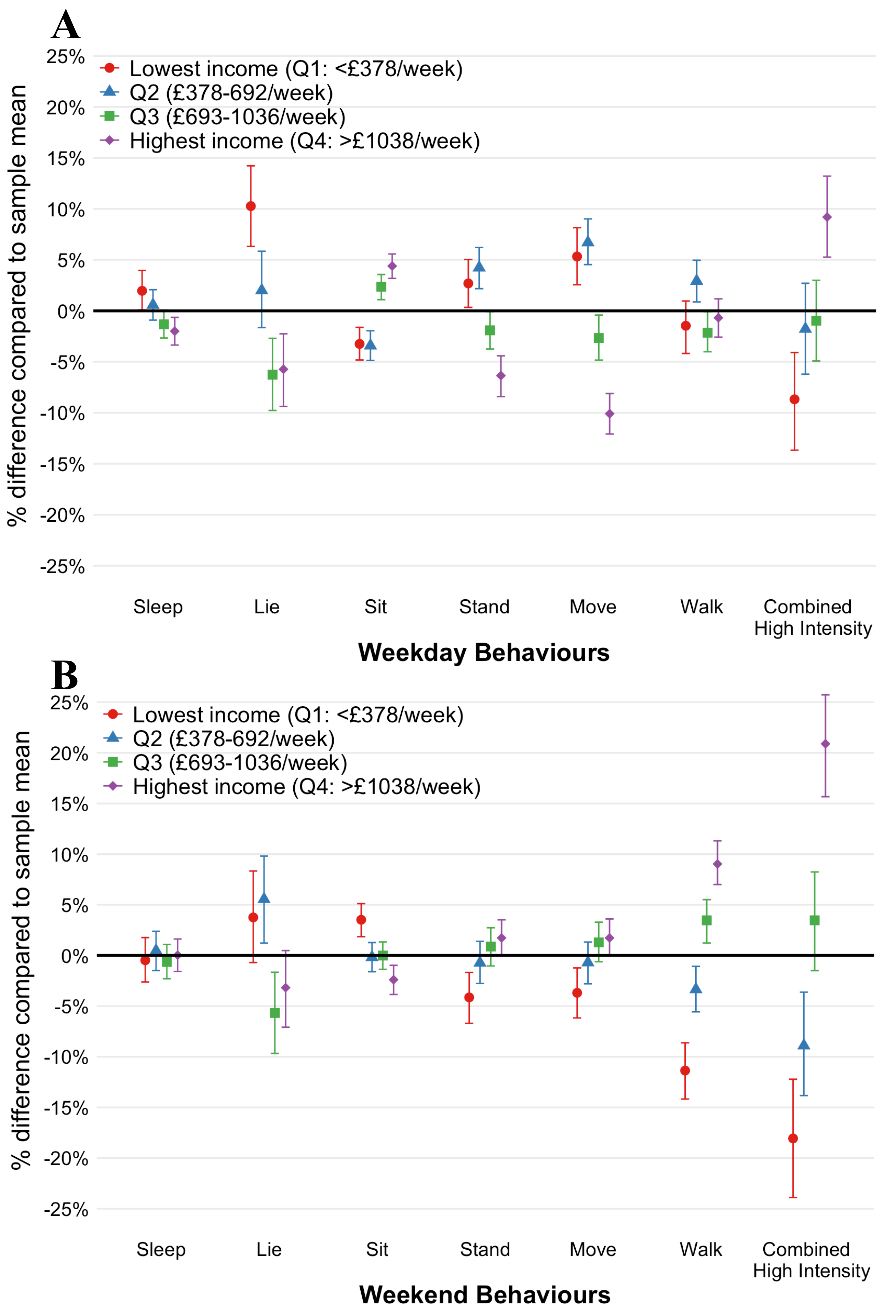

<u>Supplementary Figure 5.</u> Percent differences in daily movement between **income quartiles** compared to the sample mean on **A)** Weekdays and **B)** Weekends; derived with the log-ratio of geometric mean values for sleep, lying, sitting, standing, moving, walking, combined-high intensity physical activity, with bootstrap 95% percentile confidence intervals.

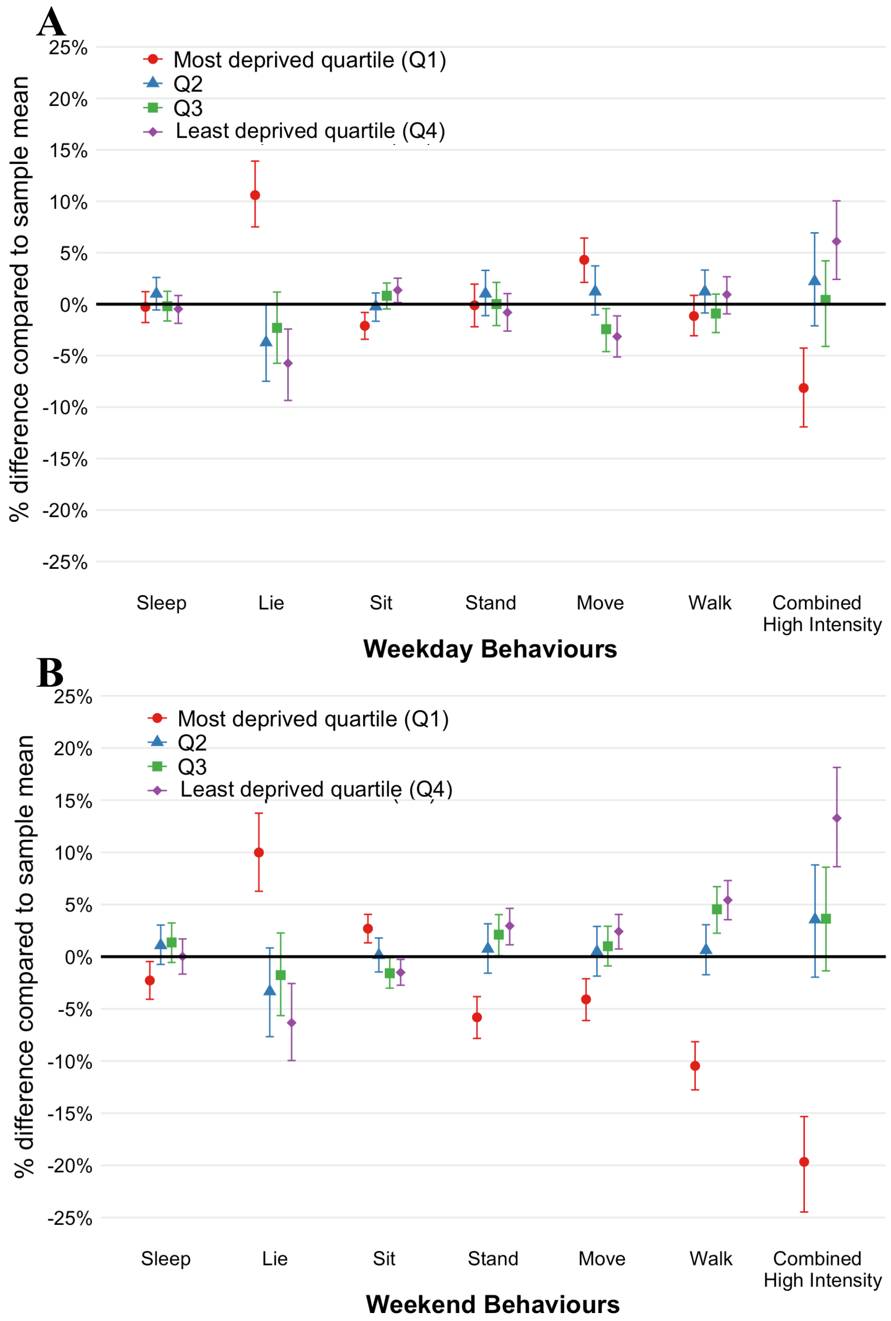

Supplementary Figure 6. Percent differences in daily movement between **Index of Multiple Deprivation quartiles classes** compared to the sample mean on **A)** Weekdays and **B)** Weekends; derived with the log-ratio of geometric mean values for sleep, lying, sitting, standing, moving, walking, combined-high intensity physical activity, with bootstrap 95% percentile confidence intervals.

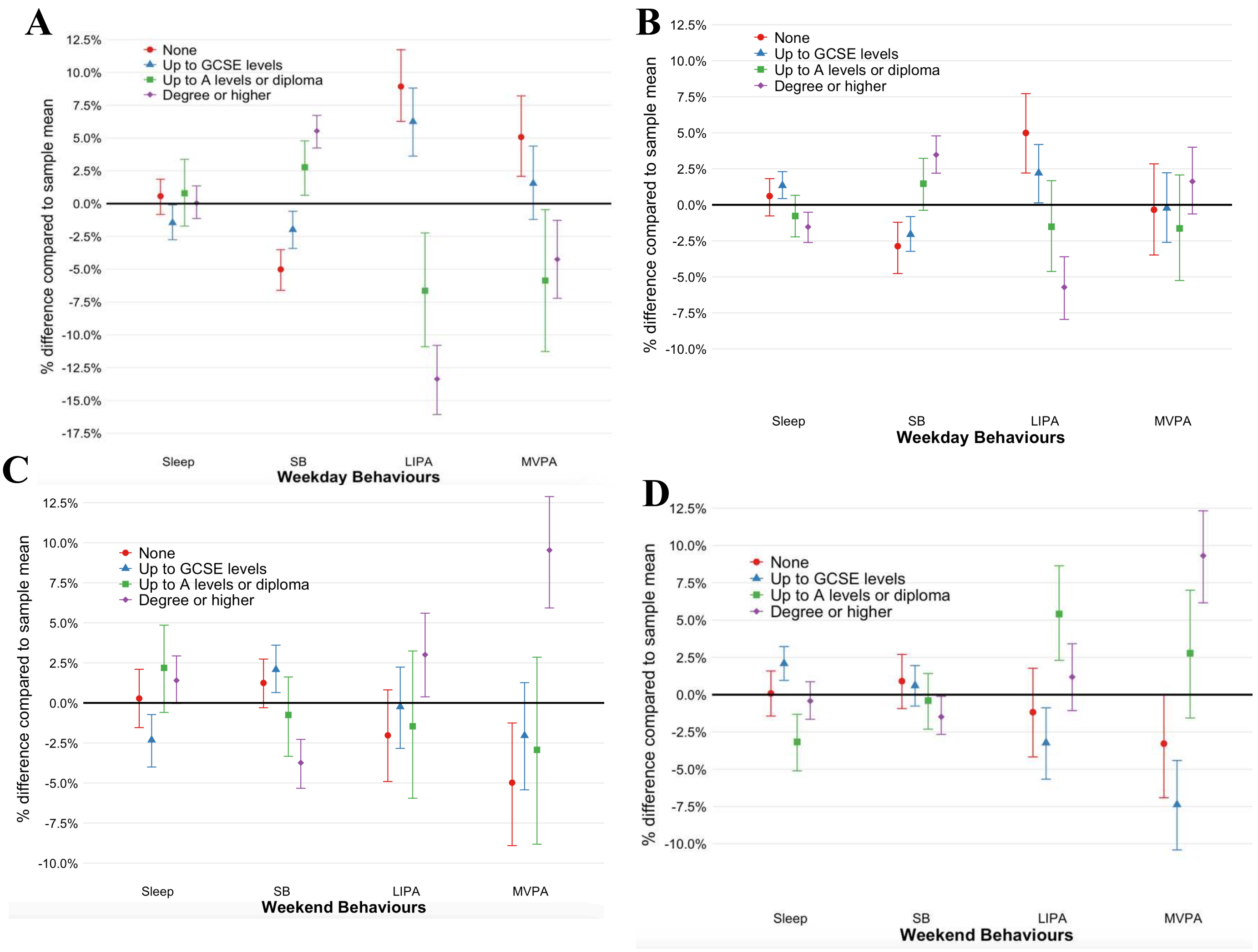

<u>Supplementary Figure 7.</u> Percent differences in daily movement between education groups compared to the sample mean in **A)** Males on weekdays; **B)** Females on weekdays; **C)** Males on weekends; **B)** Females on weekends; derived with the log-ratio of geometric mean values for sleep, sedentary behaviour, light-intensity physical activity and moderate to vigorous intensity physical activity, with bootstrap 95% percentile confidence intervals.

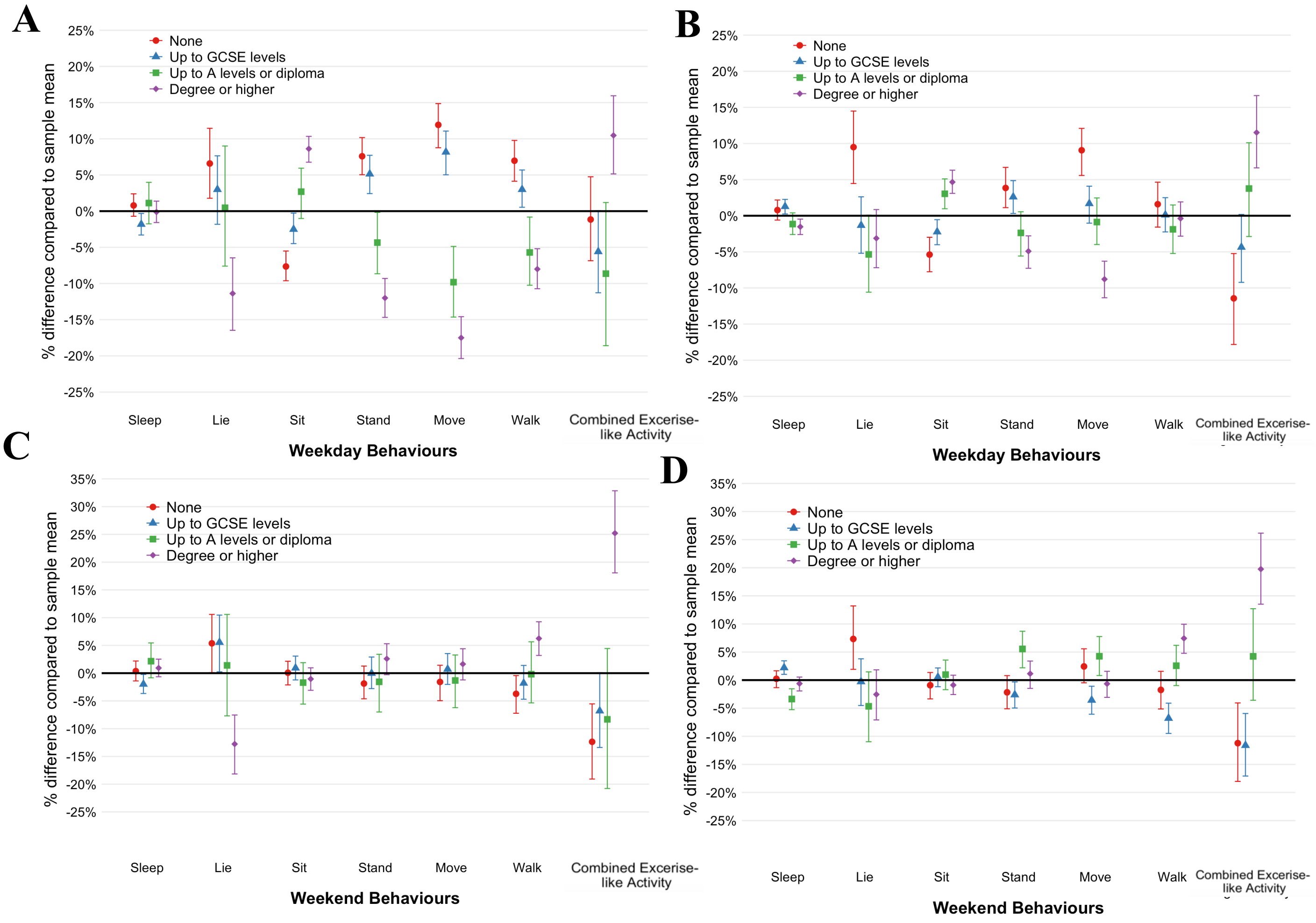

<u>Supplementary Figure 8.</u> Percent differences in daily movement between education groups compared to the sample mean in **A)** Males on weekdays; **B)** Females on weekdays; **C)** Males on weekends; **B)** Females on weekends; derived with the log-ratio of geometric mean values for sleep, lying, sitting, standing, moving, walking, combined-high intensity physical activity, with bootstrap 95% percentile confidence intervals.
